# Acute kidney stress—a useful term based on evolution in the understanding of acute kidney injury

**DOI:** 10.1186/s13054-016-1184-x

**Published:** 2016-01-22

**Authors:** Nevin Katz, Claudio Ronco

**Affiliations:** 1 Division of Cardiac Surgery, Johns Hopkins University, 1800 Orleans Street, Suite 7107, Baltimore, MD 21287 USA; 2 Foundation for the Advancement of Cardiothoracic Surgical Care (FACTS-Care), 1912 Foxhall Road, McLean, VA 22101 USA; 3 Department of Nephrology, Dialysis and Renal Transplantation, International Renal Research Institute Vicenza (IRRIV), San Bortolo Hospital, Vicenza, Italy

## Abstract

Critical care physicians have debated an appropriate term for the clinical phase preceding acute kidney injury (AKI). The recent development of cell cycle arrest biomarkers that signal the potential development of AKI is part of an evolution in the molecular diagnosis and understanding of AKI. It is proposed that the pre-injury phase that leads to AKI can be described as “acute kidney stress”. This term has the potential to expand horizons in regard to the early detection of situations that will lead to AKI and the early implementation of corrective measures.

Critical care physicians have debated an appropriate term for the clinical phase preceding acute kidney injury (AKI). “Renal angina” has been proposed [1]; however, it is recognized that AKI can result from non-ischemic mechanisms, and AKI is not associated with pain. A variety of etiologies for AKI have been identified, including sepsis, inflammation, toxins, and heart failure. Examples of complex mechanisms include the cardio-renal syndromes, which are related to interactions between the kidney and the cardiovascular system [2]. The complexity of defining AKI or a “kidney attack” using standard measures of kidney function has been recognized by nephrologists from centers around the world [3]. Another term that has been discussed is “subclinical” acute kidney injury [4].

Early diagnosis of AKI has challenged intensivists and critical care nephrologists. The delay in elevation of creatinine to approximately 24–48 h after AKI makes this standard renal function test inappropriate for the early diagnosis of AKI. Although the renal biomarker neutrophil gelatinase-associated lipocalin (NGAL) can be a valuable clinical test to alert clinicians to subclinical AKI, its lack of specificity has limited its use as a sole indicator of AKI [5]. A variety of renal biomarkers have now been identified and have the potential to enhance the understanding and diagnosis of AKI, particularly as biomarker monitoring is combined with monitoring changes in renal function [6]. The use of biomarkers in characterizing cardio-renal syndromes has been described [7]. The recent identification of cell cycle arrest biomarkers that signal the potential development of AKI is part of an evolution in the molecular diagnosis and understanding of AKI [8]. These biomarkers are released by kidney cells along a path which may lead to AKI during a pre-injury phase.

It is proposed that the pre-injury phase that leads to AKI can be described as “acute kidney stress” (AKS). The term AKS seems appropriate as the understanding of AKI evolves. It is debatable if AKS is a condition of very early injury or a condition of increased susceptibility to exposures that lead to AKI. In fact the role of the expressed biomarker molecules may represent a way to monitor an initial damage, but also an attempt of the kidney to proceed towards repair and avoid maladaptive repair and progression towards fibrosis.

Working out criteria for AKS will assist critical care teams in making clinical adjustments before AKI occurs. Potential criteria for diagnosis of AKS include indices of renal perfusion, such as urine output per minute, changes in renal cell function, and detection of biomarkers that reflect metabolic impairment, or kidney “cell cycle arrest”. The criteria in part could be based on parameters such as those used to define the first stages of AKI in the Risk, Injury, Failure, Loss of kidney function and End-stage kidney disease (RIFLE), Acute Kidney Injury Network (AKIN), and Kidney Disease Improving Global Outcome (KDIGO) criteria.

Recent developments to detect and monitor renal impairment provide innovative criteria to define AKS. It is well recognized that serum creatinine becomes elevated late in the early stages of AKI. This raises the challenge of measuring glomerular filtration rate in real time [9]. Innovative technology is being developed which may provide such measurements in the intensive care unit (ICU). To address the issue of the late elevation in serum creatinine, the measure of “renal functional reserve” has been described as a parameter of renal frailty [10]. Remarkably, in adults, technology to monitor renal blood flow has yet to be developed. Currently, urine flow is used as a surrogate to monitor renal perfusion. Areas for development include real time monitoring of renal cell perfusion. The ADQI XIII Work Group has addressed the challenge of monitoring for conditions creating renal ischemia [11].

AKI following major cardiac surgery remains an important source of patient morbidity and mortality. With the increasing complexity of cardiac surgical cases, this complication is being recognized with increased frequency at major centers around the world [12]. An important cause of cardiac surgery-associated AKI is thought to be inadequate blood flow to the kidney while the patient is on cardiopulmonary bypass and in the ICU due to suboptimal management of hemodynamics. Over the years, development of management protocols has been impeded by lack of technology to directly measure blood flow to the kidney. The use of cerebral oximetry to detect blood pressure excursions below the cerebral autoregulation threshold represents an innovative technology to alert physicians to hemodynamics that may result in AKI [13]. Such technology is now available for monitoring patients in the operating room and the ICU.

Renal ischemia related to infusion of vasoconstrictors or to local impairment of flow due to renal artery stenoses may go undetected until AKI occurs. Monitoring renal cell physiologic function for AKS could alert critical care professionals to flawed goals of hemodynamic management. Hemodynamic goals based on standard ICU guidelines, rather than physiologic monitoring, may lead to undetected renal ischemia, as individual patients may have unique arterial and/or venous pressure requirements.

Another issue in the diagnosis of AKS and its implications for long-term outcome is whether AKS may have an impact on the future development of chronic kidney disease (CKD), kidney frailty, or susceptibility to insults. Multiple AKS events may produce progressive inflammation, apoptosis, G2 cell cycle arrest, epithelial to mesenchymal transition, fibrosis and CKD. This could be a newly identified mechanism of nephroangiosclerosis (which up till today has an unknown pathogenesis).

It is anticipated that the term AKS could become increasingly useful as clinicians develop methods to diagnose and treat the early stages of AKI. This in turn should have a major impact on patient outcomes and healthcare costs. The expanded use of advanced technology, such as ventricular assist devices and extracorporeal membrane oxygenation (ECMO), to support cardio-respiratory function has led to the ICU being an extension of the operating room [14]. Use of this innovative supportive technology has been associated with increased risk of AKI [15, 16]. These developments highlight the need to define and identify the pre-AKI phase, which could be termed AKS.

Combining innovative monitoring of renal function with identification of new biomarkers of kidney stress and injury could lead to the early diagnosis of AKS, and the implementation of corrective hemodynamic and pharmacologic management. An important issue when renal impairment is occurring is timing the initiation of renal replacement therapy. A score has been developed to assist with this decision [17].

A focus on the metabolic mechanisms of AKS and the associated impairments of renal cell function has the potential to lead to innovative monitoring technology and protocols. The result would be earlier changes in clinical care to prevent kidney cell injury. The importance of interventions to prevent or reduce the extent of AKI is highlighted by the recognition that AKI is associated with the development of CKD and its associated mortality [18].

## Abbreviations

AKI: acute kidney injury
AKS: acute kidney stress
CKD: chronic kidney disease
ICU: intensive care unit
